# Symptom assessment in the dying: family members versus healthcare professionals

**DOI:** 10.1136/spcare-2023-004382

**Published:** 2023-11-16

**Authors:** Lisa Martinsson, Margareta Brännström, Sofia Andersson

**Affiliations:** 1 Department of Radiation Sciences, Oncology, Umeå University, Umeå, Sweden; 2 Department of Nursing, Campus Skellefteå, Umeå University, Umeå, Sweden

**Keywords:** Symptoms and symptom management, Supportive care

## Abstract

**Objectives:**

Symptom management and support of the family members (FMs) are considered essential aspects of palliative care. During end of life, patients are often not able to self-report symptoms. There is little knowledge in the literature of how healthcare professionals (HCPs) assess symptoms compared with FMs. The objective was to compare the assessment of symptoms and symptom relief during the final week of life between what was reported by FMs and what was reported by HCPs.

**Methods:**

Data from the Swedish Register of Palliative Care from 2021 and 2022 were used to compare congruity of the assessments by the FMs and by HCPs regarding occurrence and relief of three symptoms (pain, anxiety and confusion), using Cohen’s kappa.

**Results:**

A total of 1131 patients were included. The agreement between FMs and HCPs was poor for occurrence of pain and confusion (kappa 0.25 and 0.16), but fair for occurrence of anxiety (kappa 0.30). When agreeing on a symptom being present, agreement on relief of that symptom was poor (kappa 0.04 for pain, 0.10 for anxiety and 0.01 for confusion). The trend was that HCPs more often rated occurrence of pain and anxiety, less often occurrence of confusion and more often complete symptom relief compared with the FMs.

**Conclusions:**

The views of FMs and HCPs of the patients’ symptoms differ in the end-of-life context, but both report important information and their symptom assessments should be considered both together and individually. More communication between HCPs and FMs could probably bridge some of these differences.

**What was already known?**

- During end- of- life, symptoms and symptom ratings are done by proxies.

**What are the new findings?**

- For patients who were reported to have pain, anxiety and confusion, the healthcare professionals reported complete symptom relief more often than the family members.

**What is their significance?**

- Symptom ratings by family members and healthcare professionals differ and cannot be used fully interchangeably.
- More communication can probably bridge some of these different views.

## Introduction

 Symptom management and support of the patient and family members (FMs) are considered essential aspects of palliative care. FMs are especially important in the palliative care field, and they often have a central role during end-of-life care. When a patient is cared for in their own home, the presence of FMs is almost a prerequisite and partly what the healthcare profession expects. When a patient suffers from symptoms, such as breathlessness or depression, it burdens the FMs.^1^ When it comes to information from the healthcare professionals (HCPs) about the patient being in the end-of-life stage, it is more often the FMs who receive this information than the patients themselves. Such information has been shown to be provided to 70% of FMs, compared with 43% of patients.^2^ A probable explanation is that the patient’s disease or fragile state hinders such communication with the HCP.

Since pain and other suffering is subjective, the patient’s own self-reported symptom data are considered the most reliable information, but patients are often not able to self-report symptoms during their last days of life and the only potential data sources are FMs and HCPs. We have found few studies describing symptoms for patients with cancer assessed by HCPs compared with assessments by FMs.^3 4^ In a medical oncology hospital ward setting, Bertocci *et al*,^3^ showed that FMs and HCPs have low congruity when assessing the patient’s end-of-life care. They however also showed that HCPs play an important role in complementing data when FMs did not provide the information. Cheng *et al*,^4^ on the other hand, showed good agreement between physicians and care givers when assessing different quality of dying for patients dying from cancer, with the exception of psychological aspects. In a meta-analysis from 2017, Robertson *et al*,^5^ argued that family and staff members make similar assessments of quality of life in a dementia care home setting. Tanghe *et al*^6^ showed that HCPs rated comfort when dying for people with dementia higher compared with FMs, indicating that there might be more differences in ratings when it comes to the end-of-life care and dying setting. McPherson and Addington-Hall^7^ have argued against the common approach to consider self-reported patent data as the ‘gold standard’ and instead propose that self-reported and proxy ratings should be examined independently.

The Swedish Register of Palliative Care (SRPC) is a database about end-of-life care quality focusing on the last week of life. Data from the SRPC have previously shown that old age is a risk factor for receiving less high-quality care during end-of-life,^8 9^ and that persons with dementia receive poorer care quality during end of life compared with persons with cancer.^10^ The SRPC database contains information about occurrence and relief of six symptoms (pain, nausea, anxiety, breathlessness, respiratory secretion and confusion), and has been used to describe the symptom burden during the last week for patients with ALS,^11^ and with COVID-19.^12^ Data on these symptoms were reported by the healthcare profession to the SRPC.

## Objective

The objective of this register study was to compare the assessment of symptoms and symptom relief during the last week of life between what was reported by FMs and what was reported by HCPs.

## Methods

This study was based on data from the SRPC. The SRPC is a Swedish national web-based quality register with a national coverage of around 55%–60%, meaning that around 55%–60% of the approximately 90 000 yearly deaths in Sweden are reported to the register from the healthcare. Data are mainly collected using an end-of-life questionnaire (ELQ), which is answered retrospectively by HCPs after the death of a patient. The questionnaire is mostly answered by nurses and sometimes by doctors or other HCPs. The questionnaire contains around 30 questions concerning different aspects of end-of-life care quality, including information, support, symptoms and symptom management.

Since the beginning of 2021, a Family Member Questionnaire (FMQ) has been distributed by the SRPC. It is also to be answered retrospectively by a FM at some time after the death of the person. The HCP who reports information to the SRPC can also choose to invite the FM to answer the FMQ and provide them with a code. The FMQ is answered as a web-based questionnaire on the SPRC homepage and consist of around 20 questions about information, support and symptoms, including some questions that can be answered by free text. The FMQ was open for use for all healthcare units in Sweden during the study period.

All adult individuals in the SRPC database who were reported by both the HCP (using the ELQ) and by a FM (using the FMQ) at the time of data reception from the SRPC were included in the study. Since the FMQ has been in use since 1 January 2021, the identified individuals had died from that date onwards. Data were retrieved from the SRPC on 31 October 2022.

Background characteristics data (age, sex, place of death, and cause of death) were collected. Occurrence and relief of the symptoms measured by both questionnaires (pain, anxiety and confusion) were calculated as reported separately by the HCPs and the FMs. Statistical findings are described as mean, median, SDs, range (minimum–maximum) and proportions.

Answers in the ELQ and in the FMQ were compared regarding the occurrence of breakthrough symptoms (pain, anxiety and confusion) and relief of these symptoms using Cohen’s kappa with 95% CIs. All ‘Don’t know’ answers were excluded in these analyses and relief of symptoms were dichotomised into complete relief versus partial or no relief. Comparison of relief of symptoms was only performed for those who were reported to have suffered from that corresponding symptom by both the HCPs and the FMs. The kappa values were used to categorise the correlation according to Altman^13^: <0.20 poor agreement, 0.20–0.39 fair, 0.40–0.59 moderate, 0.60–0.79 good and 0.80–1.00 very good agreement.

During part of the study period (the year 2021), some patients were reported to have died unexpectedly and lacked symptom data. These patients were excluded in the analysis (n=5).

##  Ethical approval

The study was approved by the Swedish Ethical Review Authority on 28^th^ June 2022 (registration number 2022-03075-01). The study was approved by the SRPC management group on 17^th^ October 2022. 

## Results

The HCPs answered the questionnaire in a mean time of 6 days (SD 14) after the death of the patient and a median of 1 day, a maximum of 134 and a minimum of 0 days. The FMs answered the questionnaire in a mean time of 67 days (SD 53) after the death of the patient and a median of 55 days, a maximum of 547 and a minimum of 1 day. During the study period, around 40% of all invited FMs had answered the FMQ. During 2021, FMs were invited to complete the FMQ in around 3% of all deaths in Sweden.

*Background characteristics*: the sample identified 1131 patients. The mean age was 76.5 years (SD 12), and the median was 77 years old; half of the participants were women (50%). The most common place of death was an inpatient specialised palliative ward (42%), followed by at home with support from a specialised palliative home care team (35%). The most common cause of death was cancer (74%), followed by heart disease (16%) and dementia (8%) (table 1).

**Table 1:** Background characteristics of patients

Age years	
Mean (SD)	76.5(12)
Median	77
Range (min–max)	28–104
Sex n (%)	
Female	566 (50)
Male	565 (50)
Place of death n (%)	
Specialist palliative inpatient care	478 (42.3)
Home with support from specialised palliative home-care team	391 (34.6)
Nursing home—permanent stay/ short-term stay	181 (16)
Hospital: ward/patient facility/ICU (not hospice/palliative inpatient care)	22 (1.9)
Home with support from healthcare team without palliative specialisation	45 (4.0)
Other	14 (1.2)
Cause of death n (%)*	
Cancer	835 (73.8)
Cardiovascular disease	179 (15.8)
Dementia	91 (8.0)

* It was possible to answer multiple diagnoses as cause of death.

ICUintensive care unit

The FMs consisted of 585 husbands/wives/partners (52%), 463 children (41%), 38 siblings (3%), 24 other relatives (2%), 13 parents (1%), 7 friends (0.6%) and 1 custodian (0.1%). No further information about the FMs was available.

The most commonly reported symptom was pain, which was reported for 80% of the patients by the HCPs and for 67% by the FMs. Anxiety was reported for 63% of the patients by the HCPs and for 44% by the FMs. Confusion was the least common symptom of the three; reported for 23% of the patients by the HCPs and 42% by the FMs. The FMs more often answered ‘Don’t know’ compared with the HCPs (4% vs 1% for pain, 18% vs 3% for anxiety and 11% vs 5% for confusion; table 2).

**Table 2:** Occurrence of pain, anxiety, and confusion during the last week of life according to family members and healthcare professionals

	Family members N (%)	Healthcare professional N (%)
Pain		
Yes	757 (67)	906 (80)
No	332 (29)	217 (19)
Do not know	47 (4)	8 (1)
Total	1131 (100)	1131 (100)
Anxiety		
Yes	501 (44)	710 (63)
No	435 (38)	391 (35)
Do not know	200 (18)	30 (3)
Total	1131 (100)	1131 (100)
Confusion		
Yes	471 (42)	252 (22)
No	537 (47)	820 (73)
Do not know	123 (11)	59 (5)
Total	1131 (100)	1131 (100)

For the 906 patients where HCPs had reported pain, they had reported 764 of these (84%) as being completely relieved, 131 (14%) as partly relieved and nobody as being not relieved at all. For the 756 patients who were reported to have had pain by FMs, one was excluded from the pain relief analysis because of lacking pain relief data. Of the remaining 755, 326 (43%) were reported by FMs as being completely relieved, 396 (52%) as partly relieved and 13 (2%) as not relieved at all (table 3).

**Table 3:** Relief of symptoms

	Family members N (%)*	Healthcare professionals N (%)†
Relief of pain		
Completely	326 (43)	764 (84)
Partly	396 (52)	131 (14)
Not at all	13 (2)	0 (0)
Do not know	20 (3)	11 (1)
Total	755‡	906
Relief of anxiety		
Completely	185 (37)	569 (80)
Partly	273 (54)	129 (18)
Not at all	19 (4)	4 (1)
Do not know	24 (5)	8 (1)
Total	501	710
Relief of confusion		
Completely	85 (18)	98 (39)
Partly	266 (56)	110 (44)
Not at all	57 (12)	28 (11)
Do not know	63 (13)	16 (6)
Total	471	252

* Symptom ratings by the HCPs (regardless of the ratings by the FMs)the FMs (regardless of the ratings by the HCPs. ᵇ Symptom ratings by the FMs (regardless of the ratings by the HCPs) ᶜLack of pain relief data from the FMs for one who was reported to have had pain by the FMs. This was excluded

† Symptom ratings by the HCPs (regardless of the ratings by the FMs)

‡ Lack of pain relief data from the FMs for one patient who was reported to have had pain by the FMs. This patient was excluded.

FMsfamily membersHCPshealthcare professionals

For the 710 patients where HCPs had reported anxiety, 569 (80%) were reported as being completely relieved by the HCPs. For the 501 patients where FMs had reported anxiety, 185 (37%) were reported as being completely relieved. The HCPs had reported 252 patients with confusion, out of whom 98 (39%) were reported by them as being completely relieved. The FMs had reported 471 patients with confusion, out of whom 85 (18%) were reported as being completely relieved by the FMs (table 3).

*Comparison between symptoms reported by HCPs and FMs:* when comparing the answers from the HCPs and FMs regarding symptom occurrence, the agreement was poor for pain and confusion (kappa 0.25 and 0.16) and fair for anxiety (kappa 0.30) (table 4). Agreement for relief of symptoms was poor for all three symptoms (kappa 0.04 for pain relief, 0.10 for anxiety relief and 0.01 for confusion relief) (table 5).

**Table 4:** Comparison of prevalence of symptoms reported by healthcare professionals and family membersᵃ

	*Family members N*				
*Healthcare professionals n*	*Yes*	*No*	Total	Kappa	95% CI
Pain					
Yes	660	215	875	0.25	0.19 to 0.31
No	89	112	201		
Total	749	327	1076		
Anxiety					
Yes	388	204	592	0.30	0.24 to 0.36
No	106	211	317		
Total	494	415	909		
Confusion					
Yes	145	87	232	0.16	0.10 to 0.22
No	302	425	727		
Total	447	512	959		

ᵃOnly including cases where both HCPs and FMs had answered the question about the respectively symptom with "Yes" or "No".

**Table 5:** Comparison between relief of symptoms reported by healthcare professionals and by family membersᵃ

	*Family members N*				
Healthcare professionals n	Completely	Partly/not at all	Total	Kappa	95% CIs
Relief of pain					
Completely	245	293	538	0.04	−0.02 to 0.09
Partly/not at all	38	61	99		
Total	283	354	637		
Relief of anxiety					
Completely	127	175	302	0.10	0.04 to 0.17
Partly/not at all	17	56	73		
Total	144	231	375		
Relief of confusion					
Completely	10	38	48	0.01	−0.15 to 0.17
Partly/not at all	15	59	74		
Total	25	97	122		

ᵃ Only including cases where both HCPs and FMs had reported that the patient had suffered from that corresponding symptom.

## Discussion

This study about pain, anxiety and confusion during the last week of life showed that agreement of anxiety occurrence rating was fair between FMs and HCPs, while agreement was poor for rating of pain and confusion occurrence and relief of all three symptoms. The trend was that HCPs more often rated occurrence of pain and anxiety compared with rating by FMs, but less often occurrence of confusion. For patients who were reported to have had pain, anxiety and confusion, the trend was that the HCPs reported complete symptom relief more often than the FMs did. To our knowledge, there are no other studies comparing symptoms reported by FMs and HCPs during the last week of life in a palliative care context.

The questionnaires had been answered by FMs and HCPs in this study, which is a strength, but patient-generated data are lacking and the questionnaires are answered retrospectively, possibly introducing recall bias. The time lag between the patients’ death and responses from the FMs could have introduced additional recall bias, and information about when the FMs were invited to answer the questionnaire was not available. Because of legal issues, the SRPC database does not contain much information about the FM answering the FMQ, such as age or gender. Such data would be interesting to have to better analyse the generalisability of our findings but was not possible to obtain since it is not collected by the SRPC. The questionnaire used for data collection from the healthcare has been validated,^14 15^ but the FMQ has not been scientifically validated.

Several studies have been made about the perspectives of HCPs versus FMs in intensive care units (ICUs)^16^,^18^ and nursing homes.^19^ van der Steen *et al*,^20^ showed that FMs and healthcare nurses agreed about whether the symptom burden for patients with dementia in nursing homes was high or low. However, in contrast to the findings in this study, van der Steen *et al* also showed that nurses reported lower level of symptom management of pain and anxiety.^20^ In this study, FMs and HCPs had poor agreement for most symptom ratings. A review showed evidence that the use of proxies, such as FMs and HCPs, can be reliable on observable symptoms. However, the agreement between the FMs and HCPs was poorest for symptoms such as pain and anxiety,^7^ which is congruent with our findings. Bertocci *et al*,^3^ showed that there was poor agreement between FMs, nurses and physicians in home care, other wards and other hospitals. This result was also shown in a study in two medical ICUs at academic tertiary care medical centres, where the authors compared proxy data collection between quality of dying from family caregivers compared with caregiving physicians.^16^

The FMs have important knowledge about the patients and their symptoms before the end-of-life stage. It is also important to include FMs in decisions of care and studies have shown that FMs prefer shared decision-making.^21^ In a study, the physicians rated control of pain higher than the FMs and nurses.^17^

Most of the patients included in this study were cared for within specialised palliative care. A study has reported that the patients in specialised palliative care have benefits compared with hospitals.^22^ Even considering that the patients were cared for within specialised palliative care, the results show that there was a significant difference in reported pain, anxiety and confusion between HCPs and FMs.

Based on these data, it is not possible to determine which rating is most correct or congruent with the patients’ own views or experiences. It has previously been shown that many persons dying in residential care homes are cognitively impaired, drowsy or unconscious during their last 3 days of life,^23^ which is a hinder for communication about pain and other symptoms. Based on our clinical experience, this is also true for dying patients in other settings. However, a subgroup of patients would perhaps be possible to include in such a future study to compare their views to that of the HCPs and FMs.

It has previously been shown that FMs can act as proxies to provide knowledge that is related to end-of-life care.^24^ Kutner *et al*,^25^ sought to advance understanding of the relationships among proxy and patient reports of symptom distress and quality of life between patients, nurses and family caregivers in the hospice/palliative care setting and found that patients and proxies provided similar average reports of symptom distress, both physical and psychological.^25^ Swedish data have previously been used to show that if the patients and/or the FMs had received end-of-life conversations, patients with symptoms were more often completely relieved and had more often been prescribed PRN drugs against that,^26^ indicating that communication can be a key factor for symptom management during end-of-life care. This suggests that rather than asking which proxy has the true answer, the views of both FMs and HCPs are important and should also be considered together, as well as individually, when relevant.

Future research can focus on further development of the FMQ, to find ways to collect data more widely and from a more diverse patient group. The patients’ own views during the last week in life is also an interesting topic to explore further. Our study shows that the views on symptoms between FMs and HCPs differ in this end-of-life context, and their ratings thus cannot be used fully interchangeably. Factors that can possibly explain parts of these mismatching ratings can be that the FMs better observe confusion because of their personal knowledge, while the HCPs better observe signs of physical distress such as pain. More communication between HCPs and FMs probably can bridge some of these different views.

## Data Availability

Data are available in a public, open access repository.
